# Genuine and Secure Identity-Based Public Audit for the Stored Data in Healthcare Cloud

**DOI:** 10.1155/2018/9638680

**Published:** 2018-09-18

**Authors:** Jianhong Zhang, Zhibin Sun, Jian Mao

**Affiliations:** ^1^School of Electronic and Information Engineering, North China University of Technology, Beijing 100144, China; ^2^Guangxi Key Laboratory of Cryptography and Information Security, Guilin 541004, China; ^3^School of Electronic and Information Engineering, Beihang University, Beijing 100191, China

## Abstract

Cloud storage has attracted more and more concern since it permits cloud users to save and employ the corresponding outsourced files at arbitrary time, with arbitrary facility and from arbitrary place. To make sure data integrality, numerous public auditing constructions have been presented. However, existing constructions mainly have built on the PKI. In these constructions, to achieve data integrality, the auditor first must authenticate the legality of PKC, which leads to a great burden for the auditor. To eliminate the verification of time-consuming certificate, in this work, we present an efficient identity-based public auditing proposal. Our construction is an identity-based data auditing system in the true sense in that the algorithm to calculate authentication signature is an identity-based signature algorithm. By extensive security evaluation and experimental testing, the consequences demonstrate that our proposal is safe and effective; it can efficiently hold back forgery attack and replay attack. Finally, compared with the two identity-based public auditing proposals, our proposal outperforms the two proposals under the condition of overall considering computational cost, communication overhead, and security strength.

## 1. Introduction

With the technique progress in communication filed, the amount of the generated data is going through fast growth. Many companies working on the healthcare trade increasingly make use of cloud storage services. Instead of every hospital storing and maintaining medical data in physical servers, cloud storage is becoming a popular alternative since it can offer the clients more convenient network-connection service, on-demand data storage service, and resource-sharing service.

The aging population problem urges healthcare services to make the continuous reformation so as to obtain cost-effectiveness and timeliness and furnish the services of higher quality. Numerous specialists deem that cloud-computing technique may make healthcare services good by reducing EHC (electronic-health-record) start-up costs, such as software, equipment, employee, and various license fees. These reasons will urge to adopt the relevant cloud techniques. Let us see one instance of healthcare services in which cloud technique is applied, the *Healthcare Sensor* system can automatically collect the patients' vital data of the wearable devices which are connected to traditional medical equipment via wireless sensor networks and then upload these data to “medical cloud” for storage. Another typical instance is the *Sphere of Care by Aossia Healthcare*, it is started in 2015. These cloud-based systems can automatically collect every day real-time data of users. It alleviates manual collection burden so that the deployment of the whole medical system is simplified. However, they may make healthcare providers to face many challenges in migrating all local health data to the remote cloud server, where the paramount concerns are privacy and security since healthcare administrator no longer completely deals with the security of those medical records. After medical data are stored on the cloud, they are possibly corrupted or dropped.

To make sure the intactness of the stored data in the remote server, the patients or healthcare service providers expect that the stored data integrality can be periodically checked to avoid the damage of the stored data. However, for the individuals, one of the greatest challenges is how to carry out the termly data integrality detection when the individuals have the copy of local files. Meanwhile, the method is also infeasible to conduct data integrality verification for a source-limited individual though retrieving the total data file.

To deal with the above issue, many specialists had presented a number of problem-solving methods which aim at the diverse systems and diverse security models in [1–20]. Nevertheless, most existing problem-solving methods had built on public key infrastructure (for short, PKI). As everyone knows that the PKI-based auditing proposals exist complex key management problem, data client needs to conduct key updating, key revocation, and key maintaining, and so on. Hence, the key management and certificate verification in the PKI-data auditing system will be a troublesome issue. Furthermore, PKC also needs more storage space than the individual ID since key pair (PK, sk) needs to be locally kept. For a verifier, to guarantee data integrality, it must firstly extract PKC from public key directory and then verify whether public key certificate (for short, PKC) is valid. Therefore, it also increases computation burden and communication overhead for the verifier.

In 2014, the first so-called ID-based data integrality proposal was proposed by Wang et al. [13]. Strictly speaking, their proposal is not a kind of identity-based auditing one because the algorithm to generate metadata authentication tag is not an idenity-based algorithm, but a PKC-based one. In 2015, Yu et al. put forward a generic method of constructing identity-based public auditing system by integrating the identity-based signature algorithm with traditional PDP protocols in [15]. Their research is very significant on studying the ID-based public auditing system. However, in their scheme, the algorithm to produce metablock authentication tag is still to adopt a PKI-based one. Furthermore, in the auditing phase, the auditor firstly verifies the validity of an identity-based signature on a public key *PK*, and then it executes data integrity verificationby using this public key *PK* again, which increases the computation burden of the auditor. In 2016, Zhang and Dong brought forward a novel identity-based public auditing proposal in [16]. The proposal is the identity-based public auditing system from the literal sense since their algorithm to produce metadata authentication tag is the ID-based signature algorithm. However, their scheme is shown to be insecure in Appendix.

To increase efficiency and strengthen the security of ID-based auditing protocols, in this work, a secure and efficient ID-based auditing construction is proposed. For our construction, its original contributions are as follows:

1. On the basis of the idea of homomorphic signature in ID-based setting, we devise an authentic identity-based auditing proposal of data integrality. The proposal can not only avoid the key managing, but also relieve the auditor's burden.
2. In auditing the phase, our scheme has constant communication overhead. Compared with the two schemes [15, 16], our proposal has more advantages with regard to computational cost and communication cost.
3. In the random oracle model, the proposed proposal has serious security proof, and the corresponding proof can be tightly reduced to the CDH mathematic problem.

## 2. Architecture and Security of System

In the following chapter, in order to better understand our ID-based data integrality auditing protocol (ID-DIAP, for short), we firstly give a description of the system model, and afterwards the security model of our ID-DIAP for cloud storage is defined.

### 2.1. System Architecture

**Figure 1:**
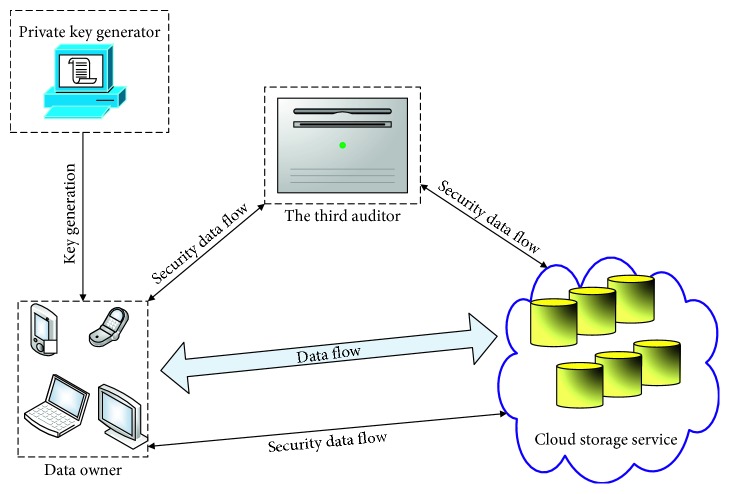
ID-based data storage model in cloud.

For our ID-DIAP system in cloud, the architecture is composed of four entities: privacy key generator (for short, the PKG), the third party verifier/auditor (for short, the TPA), cloud servers, and data user. The whole systematic architecture is demonstrated in Figure 1.

To avoid biases in the auditing process, the TPA is recommended to implement the audit function in our system model. Detail function of each role in system architecture is described as below.

- *Data User*. It acts as a cloud user and possesses a number of files which need to be uploaded to the remote cloud server without local data copy. Generally speaking, data user may be a resource-limited entity due to the limited capability of storing and computing. And it can flexibly access at any time and share the outsourced data.
- *Cloud Servers*. They are composed of a group of distributed servers and have tremendous capability of storing and computing. Furthermore, it is answerable to save and maintain the stored files in cloud. Nevertheless, the cloud server might be un-trusted, and for its own profits and a good commercial reputation, it might conceal data corruption incidents for its cloud users.
- *The Third Auditor*. It acts as a verifier of data intactness. In principle, it has professional experience and practical capability to take charge data integrality audit in the person of cloud users/data users.
- *The PKG*. It is a trusted entity and is duty bound to build up system parameters and to calculate privacy key of every cloud user.

For a cloud-based storing system, its goals are to alleviate the burden of data storage and maintaining of cloud users. Nevertheless, after data are uploaded to the remote sever in cloud, it might lead to a potential security problem since the uploaded data have been out of control for the data user and the remote server in cloud is generally unreliable. Data user might concern whether the stored files in cloud are intact. Thus, the data user wants some security measures to ensure that the integrality of the outsourced data is examined regularly without a local copy.

*Definition 1* (identity-based data integrity auditing protocol, ID-DIAP). In general, an ID-DIAP system contains the three stages:

1. *System Initialization Phase*. In this phase, the PKG is duty bound to produce system parameters. Therefore, it runs *Setup*(1^*k*^) algorithm to obtain system parameters *Para*, the PKG's key pair (*mpk*, *mpsk*) by inputting *λ* which is a safety parameter. On the contrary, the PKG also invokes KeyExtr(1^*λ*^, Para, *mpsk*, ID) algorithm to calculate privacy key *sk*_ID_ for the data user with identity ID by inputting its *mpsk* and *Para* as well as the identity ID of the data user.
2. *Data Outsourcing Phase*. In this phase, for the data owner (data user), it runs *TagGen*(*M*, sk_ID_) to generate metadata authentication tag *δ*_*i*_, on each data block *m*_*i*_ by inputting its private key *sk*_ID_ and the outsourced file *M*, where *M*=*m*_1_‖ … ‖*m*_*n*_. Finally, it uploads metadata authentication tags *δ*={*δ*_1_,…, *δ*_*n*_} to the cloud server.
3. *Data Auditing Phase*: This phase is divided into three subphases: *Challenging*, *Proof,* and *Verifying*. Firstly, the auditor runs algorithm *Challenging*(*M*_info_) to calculate *Chall* as the challenged information. After receiving *Chall*, the cloud server runs *Proof*(*M*, *δ*, and Chall) to calculate Prf as proving information, then returns Prf to the auditor. At last, the auditor invokes the algorithm *Verifying*(Chall, Prf, *mpk*, and *M*_info_) to test whether the returned proving information Prf is valid.

### 2.2. Different Types of Attack and Security Definition

In the subsection, we will analyze that our ID-DIAP system may be confronted with diverse attacks in light of the behavior of every role in the system architecture. In our system architecture, the PKG is the privacy key generator which calculates data user's privacy key. In general, it is a credible authority. We assume that the PKG does not launch any security attack to the other entities in the whole system model. For the third auditor, it is deemed to be an honest-but-curious entity and can earnestly execute every step in the auditing course. And cloud server is considered to be unreliable. It might deliberately delete or alter rarely accessed data files for saving storage space. It is a powerful inside attacker in our security model. And the goal of the attacker is to tamper and replace the stored data without being found by the auditor. Because the cloud server is a powerful attacker in our security model, we mainly consider the attacks [7] which are launched by the cloud server in this paper.

#### 2.2.1. Forge Attack

The vicious cloud server may produce a forged meta-authentication signature on a new data block or fabricate the fake proving information Prf to deceive the auditor by satisfying the auditing verification.

#### 2.2.2. Replace Attack

If a certain data block in the challenge set was corrupted, the vicious cloud server would select another valid pair (*m*_*i*_, *δ*_*i*_) of data block and authentication tag to substitute the corrupted pair (*m*_*j*_, *δ*_*j*_) of data block and data tag.

#### 2.2.3. Replay Attack

It is an efficient attack. With respect to the vicious storage server in cloud, it might produce the new proof information Prf^*∗*^ without retrieving the challenge data of the auditor though realizing the former proving information Prf.

## 3. Our Public Auditing Construction

**Figure 2:**
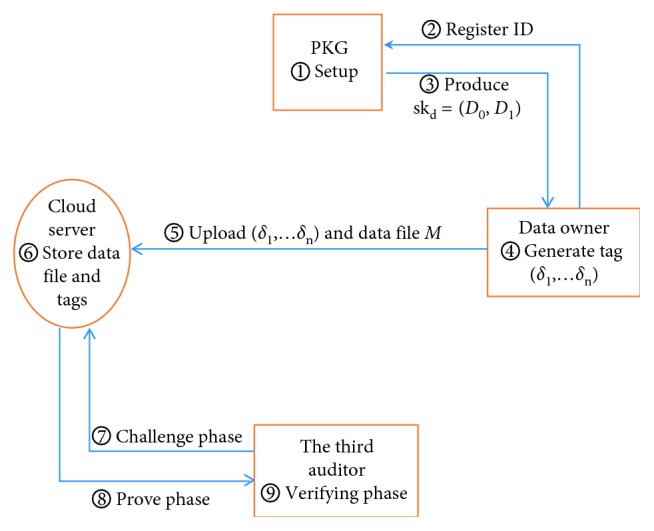
The relationship of the entities and algorithms.

In the following, we will give the description of our ID-DIAP system. It contains four entities: the PKG, cloud server, data user, and TPA. And the whole system is composed of five PPT algorithms. As for every entity and all algorithms, the diagram of the framework is represented in Figure 2. To clearly describe our protocol, the algorithms are given in detail below.

### 3.1. Setup

For the sake of enhancing readability, some notations used in our ID-DIAP system are listed in Table 1.

The PKG makes use of a parameter *λ* as an input and generates two cyclic groups *G*_1_ and *G*_*T*_. The two groups have the identical prime order *q* > 2^*k*^. And let *P*  and  *T* be two generators of group *G*_1_, where they satisfy *P* ≠ *T*. And define a bilinear pairing map *e*:  *G*_1_ × *G*_1_ → *G*_*T*_. Next, it chooses two map-to-point cryptographic hash functions *H*_0_ : {1, 0}^*∗*^ → *G*_1_ and *H*_1_ : {1, 0\}^*∗*^ → *G*_1_ and a resistant-collision hash function *H*_2_ : *G*_1_ × {1, 0}^*∗*^ → *Z*_*q*_. And the PKG randomly chooses *s* ∈ *Z*_*q*_ as its master privacy key, calculates *P*_pub_=*sP* as its public key. At last, public parameters Para are published as below:

(1) $$\begin{array}{c} \text{Para}=\left(G_{1},G_{T},q,e,P,T,H_{0},H_{1},H_{2},P_{\text{pub}}\right). \end{array}$$

And the PKG needs its master privacy key *s* to be secretly kept.

### 3.2. Key Extraction

For a data user, to produce its privacy key, it delivers its identification ID to the PKG. Subsequently, the PKG utilizes its master privacy key *s* and ID to implement the following process:

(1) Firstly, the data user delivers its identity information ID to the PKG.
(2) Next, the PKG generates (*D*_ID0_, *D*_ID1_) data user's privacy key, where
  (2) $$\begin{array}{c} D_{\text{ID}0}=sH_{0}\left(\left.\text{ID}\right\|0\right)\text{,}\\ D_{\text{ID}1}=sH_{0}\left(\left.\text{ID}\right\|1\right). \end{array}$$

And then it goes back(*D*_ID0_, *D*_ID1_) to the data user through a secret and secure channel.

(3) Upon receiving the private key (*D*_ID0_, *D*_ID1_), this data user is able to test whether its privacy key is valid through the following equations:
  (3) $$\begin{array}{c} e\left(P_{\text{pub}},H_{0}\left(\left.\text{ID}\right\|0\right)\right)=e\left(P,D_{\text{ID}0}\right),\\ e\left(P_{\text{pub}},H_{0}\left(\left.\text{ID}\right\|1\right)\right)=e\left(P,D_{\text{ID}1}\right). \end{array}$$

### 3.3. TagGen Phase

For the upload data file *M*, firstly data file *M* is divided into *n* blocks by the data user, namely, *M*=*m*_1_‖*m*_2_‖ … ‖*m*_*n*_. To outsource this file *M* to the cloud, the data user needs to randomly choose a pair (*x*_ID_, *Y*_ID_) which is a private-public key pair of a secure signature algorithm ∑=(Sig, Ver), for example, BLS short signature. Let *Name* denote the identifier of data file *M*, and then it calculates the file authentication tag *τ*=*τ*_0_‖∑·Sig(*x*_ID_, *τ*_0_), where ∑·Sig(*x*_ID_, *τ*_0_) denotes a secure signature on *τ*_0_, and *τ*_0_ denotes an information string *τ*_0_=“Name‖*n*”.

Subsequently, the data user needs to generate metadata authentication tag on the data block. To compute block authentication tags on all data blocks {*m*_*i*_}  *i*=1, 2,…, *n*, the data user uniformly samples *r* ∈ *Z*_*q*_ to calculate *R*=*rP*.

Next, for *i*=1  to  *n*, it calculates metadata authentication tag for data block *m*_*i*_ by the following steps:

(1) First of all, it calculates
  (4) $$\begin{array}{c} h_{i}=H_{2}\left(\left.{\text{Index}}_{i}\right\|\left.\text{ID}\right\|\left.R\right\|0\right). \end{array}$$
(2) And then, it makes use of its private key (*D*_ID0_, *D*_ID1_) to compute

(5) $$\begin{array}{c} \delta_{i}=rH_{1}\left(\left.{\text{Index}}_{i}\right\|\text{Name}\right)+h_{i}D_{\text{ID}0}+m_{i}D_{\text{ID}1}. \end{array}$$

(3) For data block *m*_*i*_, the resultant authentication tag of the data block is *θ*_*i*_=(*R*, *δ*_*i*_).

At last, the data user needs to upload all the meta-authentication tags (*τ*, *R*, {*δ*_*i*_}  {*i*=1, 2,…, *n*}) and the outsourced file *M* to the remote server in cloud.

On obtaining all the aforementioned data (*τ*, *R*, {*δ*_*j*_}  {*j*=1, 2,…, *n*}), the cloud server needs to execute the following validation procedure:

For *i*=1  to  *n*, it verifies the relation

(6) $$\begin{array}{c} \text{e}\left(\delta_{i},P\right)=e\left(H_{1}\left(\left.{\text{Index}}_{i}\right\|\text{Name}\right),R\right)e\left(h_{i}Q_{0}+m_{i}Q_{1},P_{\text{pub}}\right), \end{array}$$

where *Q*_1_=*H*_0_(ID‖1) and *Q*_0_=*H*_0_(ID‖0). If all relations hold, then it parses *τ* into *τ*_0_ and ∑·Sig(*x*_ID_, *τ*_0_) and verifies the validity of signature

(7) $$\begin{array}{c} \sum \cdot \text{Ver}\left(\sum \cdot \text{Sig}\left(x_{\text{ID}},\tau_{0}\right),\tau_{0},Y_{\text{ID}}\right)\;=\;?=1. \end{array}$$

If it is also valid, then the cloud server preserves these data in cloud.

### 3.4. Challenge Phase

To audit the integrality of the outsourced file *M*, firstly, the TPA parses *τ* into *τ*_0_ and ∑·Sig(*x*_ID_, *τ*_0_) and verifies ∑·Ver(∑·Sig(*x*_ID_, *τ*_0_), *τ*_0_, *Y*_ID_)=1. If it does not hold, then terminate it. Otherwise, it retrieves the corresponding file identifier name and block size *n*.

Later on, the auditor picks a subset *I*⊆[1, *n*] randomly where |*I*|=*l* and *ρ* ∈ *Z*_*q*_ to generate a challenge information

(8) $$\begin{array}{c} \text{Chall}=\left\{\rho ,I\right\}. \end{array}$$

Finally, it delivers Chall to the cloud server as the challenge.

### 3.5. Proving Phase

After obtaining the corresponding challenge information Chall={*I*, *ρ*}, for *i* ∈ {1,…, |*I*|}, cloud server calculates *v*_*i*_=*ρ*^*i*^mod*q*, and then it produces a set *Q*={*i*, *v*_*i*_}_{*i* ∈ *I*}_.

Subsequently, in light of the outsourced data file *M*={*m*_1_,…, *m*_*n*_} and meta-authentication tag *θ*_*i*_ of each block, *i* ∈ {1, 2,…, *n*}, it produces as follows:

(9) $$\begin{array}{c} \delta =\underset{j\in I}{\sum }v_{j}\cdot \delta_{j},\\ \mu =\underset{j\in I}{\sum }v_{j}\cdot m_{j}. \end{array}$$

Finally, the cloud storage server goes back 3-tuple Prf=(*δ*, *μ*, *R*) to the auditor as the corresponding proof information.

### 3.6. Verifying Phase

To check the outsourced data's integrality in cloud, after receiving the responded proof information Prf=(*δ*, *μ*, *R*), the third auditor calculates as below:

(10) $$\begin{array}{c} \bar{h}=\underset{i\in I}{\sum }v_{i}\cdot h_{i},\\ \bar{H}=\underset{i\in I}{\sum }v_{i}\cdot H_{1}\left(\left.{\text{Index}}_{i}\right\|\text{Name}\right), \end{array}$$

where *h*_*i*_=*H*_2_(index_*i*_‖ID‖*R*‖0)  for  *i* ∈ *I*.

Then, it checks the validity of the following equation:

(11) $$\begin{array}{c} e\left(\delta ,P\right)=e\left(\bar{H},R\right)e\left(\bar{h}Q_{0}+\mu \cdot Q_{1}\right). \end{array}$$

If the aforementioned Equation (11) satisfies, then the TPA outputs *VerifyRes* as true; if not, it outputs *VerifyReS* as false.

## 4. Security Analysis

To show our proposal's security, we will demonstrate that our proposal is proven to be secure against the above three attacks.

**Theorem 1 .**

Assume there exists a PPT adversary Adv that is probabilistic polynomial-time attacker (for shot PPT) and can cheat the auditor using invalid proving information Prf which is forged by the adversary Adv (the dishonest cloud storage server) in a nonignorable probability *ε*, then we are able to design an algorithm $B$ that can efficiently break the CDH assumption by invoking Adv as subprogram.

*Proof*. Let us suppose that a PPT adversary {Adv} is capable to calculate a faked proving information Prf after the data blocks or metadata authentication tags are corrupted, then we are capable of constructing another a PPT algorithm *B* which is capable of breaking the CDH assumption by utilizing the adversary Adv. First of all, let a 3-tuple (*P*, *aP*, *bP*) ∈ *G*_1_ be a CDH assumption's random instance, it is hard to obtain the solution *abP*.

To show the security proof, hash function *H*_0_ in the game is regarded as random oracle, and identity ID of each data user is only made *H*_0_-query once. For *H*_1_ and *H*_2_, they only act as one-way functions. In addition, the adversary Adv is capable of adaptively issuing the queries to three oracles: {*H*_0_-oracle}, {Key-Extract oracle}, and {TagGen oracle}.

*Setup*. Choose two cyclic groups *G*_1_ and *G*_*T*_, and their orders are the same prime number *q*. The algorithm *B* firstly sets *P*_pub_=*aP* as the public key of the PKG. Let *H*_0_, *H*_1_, and  *H*_2_ be three hash functions. Finally, it sends public system parameters (*G*_1_, *G*_*T*_, *P*, *T*, *q*, *P*_pub_, *e*, *H*_0_, *H*_1_, *H*_2_) to the adversary {Adv}. And let *j*^*∗*^ ∈ {1,…, *qH*_0_} be a challenged identity index of the data user.

*H*_0_ *-Hash Oracle*. The adversary {Adv} submits a query to *H*_0_-oracle with an identity ID_*i*_. If the index of identity ID_*i*_ satisfies *i* ≠ *j*^*∗*^, then the challenger *B* picks *t*_*i*0_, *t*_*i*1_ ∈ *Z*_*q*_ randomly to set up *H*_0_(ID_*i*_‖0)=*t*_*i*0_*P*=*h*_*i*0_ and *H*_0_(ID_*i*_‖1)=*t*_*i*1_*P*=*h*_*i*1_. Otherwise, the challenger B uniformly samples *t∗*1 and *t∗*0 from *Z*_*q*_ to set up

(12) $$\begin{array}{c} h_{i0}=H_{0}\left(\left.{\text{ID}}_{i}\right\|0\right)=t_{0}^{*}bP,\\ h_{i1}=H_{0}\left(\left.{\text{ID}}_{i}\right\|1\right)=-t_{1}^{*}bP. \end{array}$$

In the end, the 5-tuple (ID_*i*_, *h*_*i*0_, *h*_*i*1_, *t*_*i*0_, *t*_*i*1_) is added in the *H*_0_-list being initially empty.

*Key Extraction Oracle*. For a key extraction query, {Adv} submits an identity information ID_*i*_ to key extraction oracle. To response it, the challenger *B* calculates the following:

1. If the identity index of ID_*i*_ satisfies *i* ≠ *j*^*∗*^, then *B* looks for 5-tuple (*h*_*i*0_, *h*_*i*1_, ID_*i*_, *t*_*i*0_, *t*_*i*1_) in the *H*_0_-list. If it exists, *B* sends *D*_*i*0_=*t*_*i*0_*aP*, *D*_*i*1_=*t*_*i*1_*aP* to the adversary Adv; otherwise, it implicitly queries a *H*_1_-Oracle with identity ID_*i*_.
2. Otherwise, *B* terminates it.

*TagGen Oracle*. If the adversary {Adv} submits 3-tuple (M, IDi, Name) to TagGen Oracle for authentication tag query, where *M*=*m*_1_‖ … ‖*m*_*n*_ and Name is the file identifier of data file *M*. To response it, the challenge *B* calculates the following:

(1) First of all, it searches the *H*_0_-list to check if IDi exists. If it is, then the corresponding 5-tuple (*h*_*i*0_, *h*_*i*1_, ID_*i*_, *t*_*i*0_, *t*_*i*1_) in the *H*_0_-list is returned. Otherwise, *B* needs to query *H*_0_-Oracle with identity information IDi.
(2) If the identity index satisfies *i*=*j*^*∗*^, then the challenge *B* aborts it. Otherwise, it produces authentication tags on data file *M* by the following process:
  (a) Firstly, for file identifier “Name,” it picks *r*_name_ ∈ *Z*_*q*_ randomly to calculate *R*_name_=*r*_name_*P*.
  (b) Next for *l*=1 to *n*, it calculates
    (13) $$\begin{array}{c} h_{il}=H_{2}\left(\left.\left.\left.{\text{Index}}_{l}\right\|\left.{\text{ID}}_{i}\right\|R_{\text{name}}\right\}\right\|0\right). \end{array}$$

And then for *l*=1 to *n*, *B* calculates data block *m*_1_'s authentication tag as

(14) $$\begin{array}{c} \delta_{il}=r_{\text{name}}\cdot H_{1}\left(\left.{\text{Index}}_{i}\right\|\text{Name}\right)+\left(h_{il}t_{i0}+m_{l}t_{i1}\right)P_{\text{pub}}, \end{array}$$

and adds (ID_*i*_, *R*_name_, {*m*_*l*_, *δ*_*il*_}{1 ≤ *l* ≤ *n*}) to the Tag list which is initially empty.

(3) Finally, it returns (ID_*i*_, *R*_name_, {*m*_*l*_, *δ*_*il*_}{1 ≤ *l* ≤ *n*}) to the adversary {Adv}.

*Output*. In the end, for a challenge information {(*i*, *v*_*i*_)}, *i* ∈ *I*, the adversary Adv outputs a fake proving information (*δ*^*∗*^, *μ*^*∗*^, *R*^*∗*^) on data user's the corrupted file *M*^*∗*^ in a nonneglected probability *ε*, where the data user's identity is ID^*∗*^. Adv wins this security game if and only if the following constraint condition holds:

1. ID^*∗*^=ID_*j*_
2. Prf′=(*δ*^*∗*^, *R*^*∗*^) can pass verification equation (11)
3. Prf′ ≠ Prf, where Prf=(*δ*, *R*, *μ*) should be a legitimate proving information for the challenge information (*i*, *v*_*i*_), *i* ∈ *I* and the data file *M* which satisfies *M*′ ≠ *M*

When the adversary Adv wins this game, then we are capable of obtaining the following:

(15) $$\begin{array}{c} e\left(\delta^{*},P\right)=e\left(\bar{H},R^{*}\right)e\left({\hat{h}}^{*}\cdot Q_{\text{ID}j0}+\mu^{*}Q_{\text{ID}j1},P_{\text{pub}}\right),\\ e\left(\delta ,P\right)=e\left(\bar{H},R\right)e\left(\hat{h}\cdot Q_{\text{ID}j0}+\mu Q_{\text{ID}j1},P_{\text{pub}}\right), \end{array}$$

where $\hat{h}=\sum_{i\in I}v_{i}H_{2}\left(\left.{\text{Index}}_{i}\right\|\left.R^{*}\right\|\left.{\text{ID}}_{j}\right\|0\right)$ and *h*^*∗*^=∑_*i*∈*I*_*v*_*i*_*H*_2_(Index_*i*_‖*R*^*∗*^‖ID_*j*_‖0) because $\hat{H}=\sum_{i\in I}v_{i}\cdot H_{1}\left(\left.{\text{Index}}_{i}\right\|\text{Name}\right)$ is computed by the verifier, and for the same data file, *R*=*R*^*∗*^ and *h*^*∗*^=*h*. Thus, we have

(16) $$\begin{array}{c} e\left(\delta -\delta^{*}\right)=e\left(P_{\text{pub}},\left(\mu -\mu^{*}\right)Q_{\text{ID}j}\right),\\ ⇓\\ e\left(\delta -\delta^{*}\right)=e\left(aP,\left(\mu -\mu^{*}\right)\left(-t_{1}^{*}\right)bP\right),\\ ⇓\\ abP=\frac{1}{\left(\mu -\mu^{*}\right)\cdot t_{1}^{*}}\left(\delta -\delta^{*}\right). \end{array}$$

It indicates that the CDH assumption is able to be broken with nonneglected probability *ε*′. Apparently, it is impossible since it is a hard problem to solve the CDH problem.

**Theorem 2 .**

For a malicious cloud server, its replay attack in our proposed auditing proposal can efficiently be resisted.

*Proof*. The proof in detail is very alike with the security proof in [16]. Hence, it is left out due to the limited space.

## 5. Performance Evaluation

To efficiently evaluate our proposal's performance, in the following part, we show that our proposal is efficient by comparing with Yu et al.'s proposal [15] and Zhang and Dong's proposal [16] in the light of computational cost and communication overhead, where Zhang and Dong's proposal [16] which is the state-of-the-art identity-based public auditing schemes in the aspect of communication overhead.

### 5.1. Computation Costs

To evaluate the computation costs of our proposal, we would like to contrast our proposal with Zhang and Dong's proposal [16] and Yu et al.'s proposal [15] since the two schemes are recent two efficient ID-based public auditing schemes. We emulate the operators adopted in the three schemes on an HP-laptop computer with an Intel-Core i3-6500 CPU at 2.4 GHz processor and 8 GB RAM and all algorithms are implemented using the MIRACL cryptography library [21, 22], which is used for the “MIRACL-Authentication Server-Project Wiki” by Certivox. We employ a Super-singular elliptic curve over field GFp, which has the 160-bit modulus *p* and a 2-embedding degree. Moreover, in our experiments, the whole statistical results are from the mean values of 10 simulation trials.

**Figure 3:**
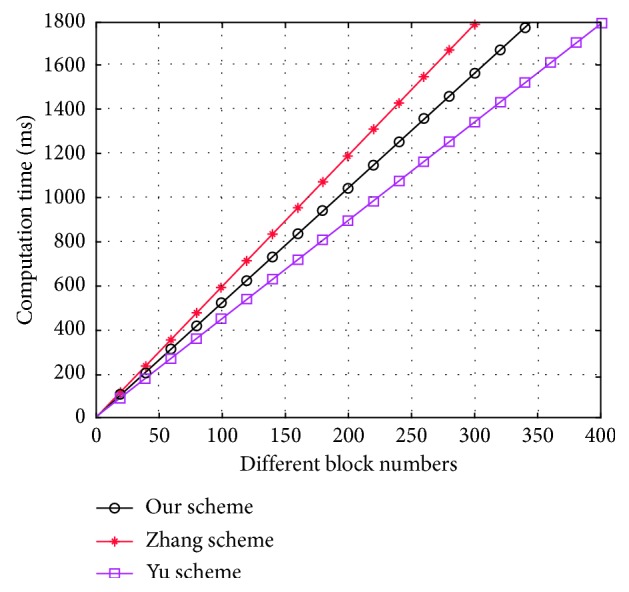
Computational cost of authentication tag generation for different block numbers.

For explicit demonstration, we use Mul_*G*1_ to denote point multiplication operation, and let *Hash* and *Pairing* be one hash-to-point operation from *Z*_*q*_ to *G*_1_ and a bilinear pairing operation, respectively. For a public auditing protocol, the computational cost in the TagGen phase is mainly determined by computation of producing block authentication tags. To outsource a data file, the data user requires (3*n* + 1) Mul_*G*1_ + *n* hash to produce block authentication tag in our construction; in Yu et al.'s proposal and Zhang et al.' s proposal, each data user needs (*n* + 1)Hash + (2*n* + 2) Mul_*G*1_ and 4*n* Mul_*G*1_ to calculate metablock authentication tag, respectively, where *n* is the numbers of data block. In Figure 3, we show the simulation result of generating the block authentication tag for the size of diverse data blocks with the identical data file.

From Figure 3, we can know that, in the TagGen phase, Zhang et al.'s proposal is the most time-consuming and Yu et al.'s proposal is the most efficient. Our proposal is slightly slower than Yu et al.'s one since the algorithm to produce the block authentication tag is a public key certificate-based signature algorithm in Yu et al.'s proposal; however, the algorithm, which is used in our proposal, is the ID-based signature algorithm. Because block authentication tags for the data file can be produced in the off-line phase, it has a little influence on the whole protocol.

In auditing the verification phase, the computational cost mainly comes from verifying proof information. It is determined by the numbers of the challenge data blocks. For our construction, to check the validity of proving information, the auditor requires 3*pairing* + *c* Hash + (*c* + 2)Mul_{*G*_1}; however, in the other two proposals, the TPA needs to execute 3*Pairing* + 2Mul_*G*1_ and 5*Pairing* + (*c* + 2)Mul_*G*1_ + *c Hash* to check the integrality of the stored file in cloud, respectively, where *c* expresses the size of the challenge subset. In Table 2, we give their comparison of computational time in the different challenge subset.

According to Table 2, we infer that the proposal in [16] is the most efficient. Our proposal is slightly more efficient than the proposal in [15]. However, the proposal in [16] is shown to be insecure, and its detail attack is shown in Appendix. At the same time, we also find that the TPA's computational costs grow linearly with the size of the challenge subset.

### 5.2. Communication Cost

In a data audit system, communication costs mainly come from two aspects. On the one hand, it is from the outsource phase of the datafile; on the other hand, it is from the auditing phase. In the outsource phase, data owner uploads data file and the corresponding meta-authentication tags. As far as our proposal, the data owner wants to upload (*n* + 1)|*G*_1| + |*M*| bits to cloud storage server; however, in the proposals [15, 16], the data owner wants to upload (*n* + 3)|*G*_1| + |*M*| bits and 2*n* · |*G*_1| + |*M*| bits, respectively. Here, |*G*_1| represents the bit length of an element of group *G*_1_, |*M*| denotes the bit length of data file, and *n* is the number of data blocks.

In the auditing phase, communication costs are mainly from the challenge information and proving information transmitting between the TPA and cloud storage servers. In our scheme, the challenge information Chall is |*Z*_*q*| + |*I*| bits, proving information is 3·|*G*_1| bits, and thus, the total communication overhead is |*Z*_*q*| + |*I*| + 3·|*G*_1| bits, where |*Z*_*q*| represents the bit length of an element in group *Z*_*q*_ and |*I*| is the size of the challenge subset. In the proposal [16], the total communication cost is 2|*Z*_*q*| + |*I*| + 2·|*G*_1| bits in the auditing phase; in the proposal [15], the total communication costs is (2 + |*I*|)|*Z*_*q*| + |*I*| + 5·|*G*_1| bits in the auditing phase. Their comparison in detail is shown in Table 3.

As shown in Table 3, our scheme has the least communication overhead among three schemes.

### 5.3. Security Comparison

According to *Theorem 1*, we know that our scheme is provably secure against the vicious cloud server in the computational Diffie–Hellman assumption, and it has tight security reduction. For Yu et al.'s proposal in [15], their proposal is also provably secure against the vicious cloud server under the CDH assumption. However, for Zhang and Dong's proposal [16], it is shown to be insecure against the vicious cloud server attack. A vicious cloud server is capable of deleting the whole file without being conscious of the TPA, and the detail security analysis is given in Appendix.

## 6. Conclusion

In this work, we present a novel identity-based public audit system by merging the homomorphic authentication technique in the ID-based cryptography into the audit system. Our proposal overcomes the security problem and efficiency problems which have existed in the ID-based public audit systems. Finally, the proposal is proven to be secure, their security is tightly relevant to the classical CDH security assumption. By compared with two efficient ID-based schemes, our scheme outperforms those two ID-based schemes under the condition of overall considering computation complexity, communication overhead, and security.

## Figures and Tables

**Table 1:** Notations in our ID-DIAP system.

Symbol	Meaning
*K*	A security parameter
*H*_0_, *H*_1_, *H*_2_	Three hash functions
*S*	The master key of the PKG
*T*	A random generator of group *G*_1_
Prf	The proof information
Chall	The challenge information
*Q*	A large prime number
*G*_*T*_	A multiplicative group with the order *q*
*G*_1_	An additive group with the order *q*
*E*	A bilinear pairing
|*I*|	The number of elements in set *I*

**Table 2:** Comparison of computation time in the auditing phase.

Scheme	The number of the challenged blocks		
	*c*=300	*c*=460	*c*=1000
Computation time in Yu et al.'s scheme (s)	0.644	0.9733	2.31
Computation time in Zhang et al.'s scheme (s)	0.109	0.161	0.333
Computation time in our scheme (s)	0.616	0.9367	2.02

**Table 3:** Comparison of communication overhead and security among three schemes.

Scheme	Challenge information (bits)	Proof information (bits)	Total (bits)	Security
Zhang et al.'s scheme	|*I*|+|*Z*_*q*_|	|*Z*_*q*_|+2|*G*_1_|	2|*Z*_*q*_|+2|*G*_1_|+|*I*|	No
Our scheme	|*I*|+|*Z*_*q*_|	3|*G*_1_|	|*Z*_*q*_|+3|*G*_1_|+|*I*|	Yes
Yu et al.'s scheme	|*I*||*Z*_*q*_|+|*I*|+2|*G*_1_|	2|*Z*_*q*_|+|*I*|+3|*G*_1_|	(2+|*I*|)|*Z*_*q*_|+3|*G*_1_|+|*I*|	Yes

## Appendix

For a vicious *s* cloud storage server, its attack is conducted as follows:

(1) Suppose that FM is an outsourced file. It is firstly partitioned into *n* blocks, i.e., FM=*m*_1_‖ … ‖*m*_*n*_. And *π*_*i*_=(*δ*_*i*_, *R*_*i*_) is a meta-authentication signature of each block *m*_*j*_, for *j*=1,…, *n*.
(2) For a vicious cloud storage cloud, it calculates *h*_*i*_=*H*_2_(*m*_*i*_‖ID‖*R*_*i*_‖0), 1 ≤ *i* ≤ *n*.
(3) Subsequently, it uniformly samples a number *r*_*a*_ ∈ *Z*_*q*_ to compute ${\hat{R}}_{i}=m_{i}R_{i}+r_{a}m_{i}P$ and ${\hat{\delta }}_{i}=\delta_{i}+r_{a}m_{i}T$ for *i*=1, 2,…, *n*. And delete all data blocks *m*_*i*_ from the cloud server for *i*=1,…, *n*.
(4) After the challenge information Chall=(*ρ*, *I*) is received; the vicious remote server in cloud firstly calculates *v*_*i*_=*ρ*^*i*^  mod  *q*  for  *i* ∈ *I*.
(5) Then, it calculates $\delta^{*}=\sum_{i\in I}v_{i}{\hat{\delta }}_{i}$, *h*^*∗*^=∑_*i*∈*I*_*v*_*i*_*h*_*i*_, and $R^{*}=\sum_{i\in I}v_{i}\cdot {\hat{R}}_{i}$. Note that *h*_*i*_ has been calculated in step 2.
(6) The forged proof information is Prf^*∗*^=(*δ*^*∗*^, *h*^*∗*^, *R*^*∗*^). Since the forged proof information satisfies the relation
  (A.1) $$\begin{array}{c} e\left(\delta^{*},P\right)=e\left(\underset{i\in I}{\sum }\left(\upsilon_{i}\delta_{i}+\upsilon_{i}r_{a}T\right),P\right)\\ =e\left(\underset{i\in I}{\sum }\upsilon_{i}\left(r_{i}m_{i}T+h_{i}D_{\text{ID}0}+{h^{\prime }}_{i}D_{\text{ID}1}\right)+\upsilon_{i}r_{a}m_{i}T,P\right)\\ =e\left(\underset{i\in I}{\sum }\upsilon_{i}\left(r_{i}m_{i}T+r_{a}m_{i}T\right),P\right)\cdot e\left(\underset{i\in I}{\sum }\upsilon_{i}\left(h_{i}D_{\text{ID}0}+{h^{\prime }}_{i}D_{\text{ID}1}\right),P\right)\\ =e\left(T,\underset{i\in I}{\sum }\upsilon_{i}\left(r_{i}+r_{a}\right)m_{i}P\right)\cdot e\left(\left(\underset{i\in I}{\sum }\left(\upsilon_{i}h_{i}\right)D_{\text{ID}0}+\underset{i\in I}{\sum }\left(\upsilon_{i}{h^{\prime }}_{i}\right)D_{\text{ID}1}\right),P\right)\\ =e\left(T,R^{*}\right)\cdot e\left(\left(\left(h^{*}D_{\text{ID}0}+{h^{\prime }}^{*}\right)D_{\text{ID}1}\right),P\right). \end{array}$$
